# Effects of Low-Carbohydrate versus Mediterranean Diets on Weight Loss, Glucose Metabolism, Insulin Kinetics and β-Cell Function in Morbidly Obese Individuals

**DOI:** 10.3390/nu13041345

**Published:** 2021-04-18

**Authors:** Domenico Tricò, Diego Moriconi, Rossana Berta, Simona Baldi, Alfredo Quinones-Galvan, Letizia Guiducci, Stefano Taddei, Andrea Mari, Monica Nannipieri

**Affiliations:** 1 Department of Surgical, Medical and Molecular Pathology and Critical Care Medicine, University of Pisa, 56126 Pisa, Italy; diego.moriconi@yahoo.com; 2 Bariatric Surgery Division, University Hospital of Pisa, 56124 Pisa, Italy; rossanaberta@gmail.com; 3 Department of Clinical and Experimental Medicine, University of Pisa, 56126 Pisa, Italy; simona.baldi@dmi.unipi.it (S.B.); stefano.taddei@med.unipi.it (S.T.); monica.nannipieri@dmi.unipi.it (M.N.); 4 Fondazione Toscana Gabriele Monasterio, 56126 Pisa, Italy; quinones@ftgm.it; 5 Institute of Clinical Physiology, National Research Council, 56124 Pisa, Italy; letiziag@ifc.cnr.it; 6 Institute of Neuroscience, National Research Council, 35127 Padua, Italy; andrea.mari@cnr.it

**Keywords:** beta cell function, glucose tolerance, high protein diet, insulin clearance, insulin secretion, insulin sensitivity, Mediterranean diet, obesity, weight loss intervention

## Abstract

Low-calorie Mediterranean-style or low-carbohydrate dietary regimens are widely used nutritional strategies against obesity and associated metabolic diseases, including type 2 diabetes. The aim of this study was to compare the effectiveness of a balanced Mediterranean diet with a low-carbohydrate diet on weight loss and glucose homeostasis in morbidly obese individuals at high risk to develop diabetes. Insulin secretion, insulin clearance, and different β-cell function components were estimated by modeling plasma glucose, insulin and C-peptide profiles during 75-g oral glucose tolerance tests (OGTTs) performed at baseline and after 4 weeks of each dietary intervention. The average weight loss was 5%, being 58% greater in the low-carbohydrate-group than Mediterranean-group. Fasting plasma glucose and glucose tolerance were not affected by the diets. The two dietary regimens proved similarly effective in improving insulin resistance and fasting hyperinsulinemia, while enhancing endogenous insulin clearance and β-cell glucose sensitivity. In summary, we demonstrated that a low-carbohydrate diet is a successful short-term approach for weight loss in morbidly obese patients and a feasible alternative to the Mediterranean diet for its glucometabolic benefits, including improvements in insulin resistance, insulin clearance and β-cell function. Further studies are needed to compare the long-term efficacy and safety of the two diets.

## 1. Introduction

The obesity pandemic and the dramatic increase in obesity-related metabolic diseases, including type 2 diabetes mellitus [1], underscore the urgent need to compare the effectiveness of widely used nutritional strategies on weight loss and glucose metabolism.

Low-calorie Mediterranean-style (Med) diets with balanced macronutrient composition are recommended by current guidelines for weight loss [2,3] and for their additional cardiometabolic benefits [4,5,6,7,8,9,10,11]. The traditional Med diet is characterized by high proportion of vegetables, legumes, fruits, grains, nuts, and olive oil, moderate consumption of fish and red wine, and reduced intake of whole-fat dairy products, red meat, and processed foods [12,13]. Prospective studies have shown a reduced risk of developing diabetes [10,14] and a lower need for glucose-lowering medications for diabetes management [11] in people following a Med diet. In the Dietary Intervention Randomized Controlled Trial (DIRECT) [6], a Med-style diet induced a more sustained weight loss compared with a low-fat diet with similar calorie restriction. Furthermore, in a subgroup of participants with type 2 diabetes, the Med diet reduced fasting plasma glucose, insulin and markers of insulin resistance at 24 months to a greater extent compared with the low-fat diet.

A low-carbohydrate (LC), high-protein diet has been proposed as a feasible alternative to Med diets, particularly in the short term, for its greater ability to reduce body weight [6,15,16,17,18] and inhibit weight regain [19], to maintain lean body mass [20,21], to induce satiety and suppress hunger [22], to reduce liver fat content [23], and to increase thermogenesis [20,24,25]. However, studies comparing the efficacy of LC diets with standard eating patterns on weight loss and glucose metabolism yielded conflicting results [18,21,25]. During the first 6 months of the DIRECT study [6], subjects on a LC diet without calorie restriction achieved a 40–50% greater weight loss compared with the calorie-restricted Med and low-fat diets. However, they tend to regain weight at 12 months during the weight maintenance phase, reaching a plateau towards the mean weight change observed with the Med diet. Moreover, 24-month changes in plasma insulin and insulin resistance were numerically greater in non-diabetic subjects following the LC diet, but smaller in patients with type 2 diabetes, compared with the Med diet.

Along with the improvement in insulin resistance associated with weight-loss, an amelioration of β-cell function may contribute to the glucometabolic benefits of the diet. In fact, both the Med diet [26] and LC diet [25] produced a greater increase in surrogate markers of β-cell function, compared with the respective control diets, in insulin-resistant subjects without diabetes. Previous studies on β-cell function however are limited by the poor characterization of insulin kinetics, which requires the implementation of C-peptide deconvolution methods to estimate insulin secretion and to dissect the separate contribution of insulin clearance on the resulting plasma insulin levels.

The aim of this study was to compare the effectiveness of a calorie-restricted LC diet with a Med diet on weight loss and on the main determinant of glucose homeostasis in morbidly obese, insulin-resistant individuals at high risk to develop diabetes. To overcome the limitation of previous studies, insulin secretion, insulin clearance, and the different components of β-cell function were estimated by modeling plasma glucose, insulin and C-peptide profiles during 75-g oral glucose tolerance tests (OGTT) performed at the beginning and at the end of each dietary intervention.

## 2. Materials and Methods

### 2.1. Study Participants

Thirty-six morbidly obese patients on the waiting list for bariatric surgery were recruited from the outpatients’ clinic for metabolic diseases and bariatric surgery of the University Hospital of Pisa in 2018–2019. Main inclusion criteria were a BMI equal or higher than 35 kg/m^2^ (obesity grade II–III), weight stable (±1%) for at least 6 months prior to the study, age between 25 and 60 years, both women and men. We excluded patients with known diabetes, chronic kidney disease, heart failure, liver failure or NASH, chronic diarrhea (including inflammatory bowel disease), endocrine diseases including secondary causes of obesity, or taking medications known to affect glucose metabolism or body weight. All participants had a detailed medical history and a complete physical examination. The study was conducted according to the guidelines of the Declaration of Helsinki and approved by the Institutional Ethics Committee. Informed written consent was obtained from all subjects before enrollment.

### 2.2. Study Protocol

**Figure 1:**
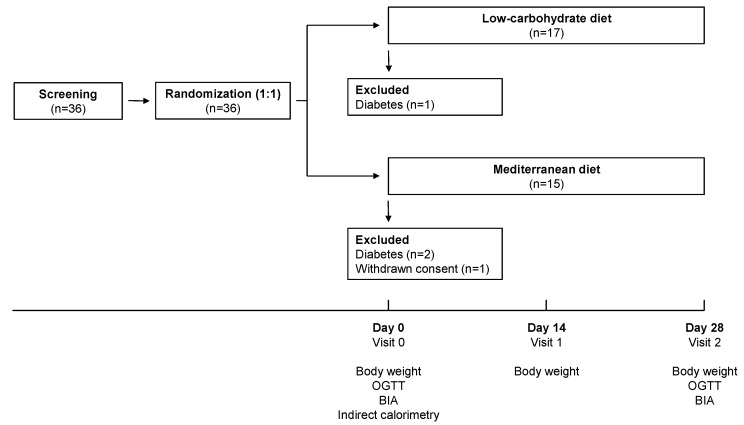
Study design. BIA, bioimpedance analysis; OGTT, oral glucose tolerance test.

In this parallel-arm, open, randomized clinical trial, patients were randomly assigned to two types of low-calorie diets designed by a qualified dietician for 4 weeks: a LC diet and a Med diet (Figure 1). All participants underwent two sessions of behavioral dietary counseling. At randomization, the session focused on nutrition education and dietary errors to avoid, with specific recommendations for each type of diet. The second visit was performed after 2 weeks to verify dietary adherence and reinforce nutritional recommendations, reducing the prescribed daily calorie intake in case of inadequate weight loss. At the beginning and at the end of each dietary intervention, subjects were admitted to our Clinical Research Unit for metabolic assessments.

### 2.3. Dietary Interventions

The LC diet was primarily made of beef, veal, cold cuts (carpaccio, cured ham), eggs and seasoned cheese (e.g., parmesan), vegetables and fruits. The energy distribution of macronutrients was 30% carbohydrate, 30% protein and 40% fat. The Med diet was rich in whole grains (pasta, bread, whole wheat), eggs, poultry, fish, vegetables, legumes, fruits and olive oil as the main condiment, and low in red and processed meat, according to the Mediterranean-style diet pyramid [12]. The energy distribution of macronutrients was 55% carbohydrate, 15% protein and 30% lipids. A detailed dietary plan containing food to be favored or avoided was provided to each participant, including a table of possible substitutions with variable equicaloric amounts of foods. The proposed food substitutions were designed to keep the daily macronutrient intake within the desired macronutrient ratio. The daily calorie intake was tailored on each patient by calculating a 50% energy deficit from the Resting Energy Expenditure (REE) derived from the indirect calorimetry.

### 2.4. OGTT

At baseline and after 4 weeks of diet, participants were admitted to our Clinical Research Unit at 08:00 a.m. after an overnight fast (10–12 h) to undergo a 75 g OGTT. A 20-gauge polyethylene cannula was inserted into a wrist vein for blood sampling, and the forearm was kept wrapped into a heated blanket to achieve arterialization of venous blood. After two baseline blood samples were drawn, participants consumed an oral glucose drink consisting of 150 mL of 50% dextrose solution (*w*/*v*) within 5 min. Timed arterialized blood samples were collected at times −15, 0, 15, 30, 45, 60, 90, 120, 150 and 180 min during the test to measure plasma glucose, insulin, C-peptide, GLP-1, and GIP. Plasma glucose was measured immediately by the glucose-oxidase technique (Beckman Glucose Analyzer II, Fullerton, CA, USA). Blood samples were centrifuged for 15 min (3000× *g* at 4 °C) and frozen at −20 °C before analysis. Insulin and C-peptide measurements were performed by electrochemiluminescence on a COBAS e411 instrument (Roche, Indianapolis, IN, USA). Plasma GLP-1 and GIP were assessed by multiplex immunoassays (Milliplex^®^ Map kit, Merck KGaA, Darmstadt, Germany).

### 2.5. Body Composition

Body composition was determined by conventional bioimpedance analysis (BIA) with a single frequency (0.4 mA, 50 KHz) electrical impedance plethysmograph (EFG-Akern, Firenze, Italy) according to the standard tetrapolar technique, in patients lying in supine position. Two electrodes were placed on the dorsal surface of the right wrist and hand, and two on the dorsal surface of the right foot. Patients were evaluated after an overnight fasting, after emptying the bladder. Body composition was calculated from the values of resistance and reactance combined with anthropometric data by applying the software provided by the manufacturer, which incorporated validated predictive equations for total body water, fat mass and fat-free mass. Waist circumference was measured at the narrowest circumference between the lower rib margin and anterior superior iliac crest. Hip circumference was measured around the widest portion of the buttocks, and the waist/hip ratio was calculated.

### 2.6. Indirect Calorimetry

Resting Energy Expenditure (REE) was measured using indirect calorimetry (Vmax Series 29, SensorMedics, Yorba Linda, CA, USA). The parameters used for its calculation were the oxygen consumed and the carbon dioxide produced, according with the modified Weir formula [27]. Before measurement, the equipment was calibrated, controlling the environmental temperature (22–23 °C), to allow thermal homeostasis. The test was performed between 8:00 and 9:00 a.m. after an overnight fast. During calorimetry, patients were awake and in the supine position. The calorimetry lasted for at least 20 min after achieving steady state.

### 2.7. Mathematical Modelling and Calculations

Insulin sensitivity was estimated by the homeostasis model assessment (HOMA) index [28] and the oral glucose insulin sensitivity (OGIS) index [29].

Insulin secretion rate (ISR) was estimated via C-peptide deconvolution using the Van Cauter’s model of C-peptide kinetics [30].

Endogenous insulin clearance, which largely reflects hepatic insulin clearance, was calculated as the ratio between fasting ISR and plasma insulin levels (ISR_fast_/I_fast_) and as the ratio of their areas under the curve (AUC) calculated by the trapezoidal rule over the duration of the OGTT (ISR_AUC_/I_AUC_) [31,32].

The different parameters of β-cell function were calculated by modelling insulin secretion and glucose concentration, as previously reported in detail [33,34]. Briefly, this model describes insulin secretion as the sum of two components. The first represents the dependence of insulin secretion on absolute glucose concentration. The quasi-linear dose–response function relating the two variables is described by a slope and an intercept, named β-cell glucose sensitivity and ISR at 5 mmol/L glucose (ISR@5), respectively, and is modulated by a time-dependent factor termed potentiation. The second component represents the dependence of ISR on the rate of change of glucose concentration and is named β-cell rate sensitivity.

### 2.8. Statistical Analysis

Continuous variables are presented as means ± standard deviations (SD) and nominal variables are reported as counts and/or percentages. Variables with a skewed distribution are presented as median [interquartile range]. Baseline differences between groups were tested by Mann Whitney test or Fisher’s exact test, as appropriate. Repeated measures were analyzed by multivariate analysis of variance (MANOVA). The *p*-values for differences between groups (diet), between time-points (time), and of the effect of the interaction between diet and time are reported. Statistical tests were performed using JMP Pro 14.3.0 (SAS Institute Inc., Cary, NC, USA) using a two-sided α level of 0.05.

## 3. Results

### 3.1. Study Participants

Thirty-six morbidly obese patients attended for a screening visit, all of whom were randomized to the LC diet or Med diet (Figure 1). One participant withdrew before beginning the intervention and 3 were excluded for the presence of unknown diabetes at the baseline OGTT, diagnosed according to the current guidelines [35]. Thirty-two (88.9%) participants completed the study and were included in the analysis, of whom 17 (53.1%) in the LC-group and 15 (46.9%) in the Med-group. Baseline clinical and metabolic characteristics were similar between the two groups (Table 1).

### 3.2. Body Weight and Composition

Overall, a 4.8% weight loss was achieved (range 2.0% to 7.7%), with a BMI reduction of 2.36 kg/m^2^ (range 0.87 kg/m^2^ to 4.01 kg/m^2^). The average weight loss was 58% greater in the LC-group compared with the Med-group (5.7 ± 1.8% and 3.6 ± 1.6%, respectively; *p* = 0.001), with similar changes in waist circumference, fat mass, and basal metabolic rate (Table 2).

### 3.3. Glucose Tolerance and Insulin Sensitivity

Fasting plasma glucose and glucose tolerance were not affected by the diet (Table 3). Fasting plasma insulin was similarly reduced by the two diets while mean insulin levels during the OGTT were unchanged. Consistently, there was a significant improvement in insulin resistance under fasting conditions, as measured by the HOMA-IR, without significant improvements during the OGTT, as measured by the OGIS index.

### 3.4. B-Cell Function and Insulin Clearance

**Figure 2:**
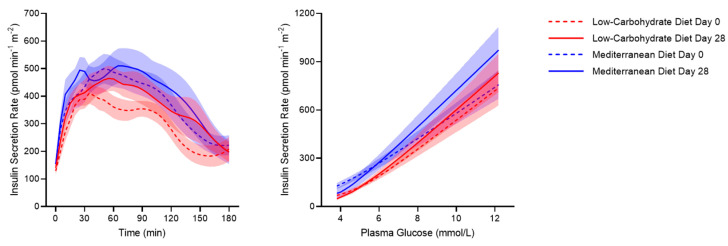
Insulin secretion rate during a 75-g OGTT (**left** panel) and insulin secretion rate against plasma glucose levels (**right** panel) in morbidly obese individuals before (dotted lines) and after (continuous lines) a 4-week low-carbohydrate diet (red lines) or Mediterranean diet (blue lines). Thick lines indicate mean and shaded areas indicate SEM.

Baseline and glucose-stimulated insulin secretion estimated by C-peptide deconvolution were similarly increased by the dietary interventions (Figure 2 and Table 3). Among model-derived parameters of β-cell function, the β-cell glucose sensitivity showed a similar improvement after the two diets, whereas incretin hormones were reduced. Similar or even lower peripheral insulin levels despite enhanced insulin secretion were explained by a greater fasting and total insulin clearance, whose increase tended to be greater after the LC diet compared with the Med diet (fasting: +16.1 [137.6] % and +8.5 [37.7] %, respectively, *p* = 0.06; total: +21.4 [81] % and −3.0 [33.1] %, respectively, *p* = 0.06).

## 4. Discussion

In this study involving morbidly obese, insulin-resistant individuals at high risk to develop type 2 diabetes, we demonstrated that a LC/high-protein diet is a feasible alternative to a Med diet with balanced macronutrient composition for weight loss and glucose management. The subjects on LC diet exhibited greater absolute and percentage body weight reduction than the Med diet, despite same daily calorie restriction. The two dietary regimens proved similarly effective in improving insulin resistance and fasting hyperinsulinemia, which are key pathogenetic mechanisms of diabetes progression [36,37,38], while enhancing β-cell function and endogenous insulin clearance.

The subjects on LC diet achieved a ~60% greater weight loss than the Med diet, to an extent (>5%) that has proven effective in decreasing diabetes incidence and improving cardiovascular risk factors in obese patients [2]. Compared with standard dietary regimens, a LC/high-protein diet has generally demonstrated greater effectiveness in reducing body weight in the short term [6,15,16,17,18], which was attributed to the greater ability of protein to induce satiety and suppress hunger [22] and to increase thermogenesis [20,24,25] than carbohydrate and fat. In fact, in morbidly obese patients eligible to bariatric surgery, a LC diet is recommended as a feasible approach to obtain a 5–10% weight loss in the immediate preoperative period, which facilitates surgery and reduces the risk of complications [39]. The higher efficacy of LC diets on weight loss and maintenance was not consistently reported in longer-term studies conducted over a 6- to 24-month period [18,21,25]. Therefore, combined nutritional approaches have been proposed implementing LC diets to achieve a rapid weight loss followed by Mediterranean-style dietary regimens for long-term weight maintenance [40].

Plasma glucose levels at fasting and in response to the oral glucose ingestion were not substantially affected by the two diets. There was however a significant improvement in insulin sensitivity in fasting conditions, likely related to the weight loss, which translates into the lower need for insulin to obtain the same glucose levels after the dietary intervention. These findings are mostly in agreement with previous studies testing similar dietary approaches. In the DIRECT study [6], a calorie-restricted Mediterranean-style diet reduced plasma insulin and markers of insulin resistance at 24 months, without improvements in fasting glucose and glycated hemoglobin except in participants with type 2 diabetes. In insulin resistant women, a 6-month LC/ high-protein diet without calorie restriction yielded a greater reduction in body weight compared with the standard dietary prescription, which again occurred without significant improvements in glucose levels [18]. Furthermore, a randomized clinical trial in overweight and obese individuals comparing four low-calorie diets with different macronutrient intake observed a larger decrease in fasting insulin levels with high-protein diets (protein 25%) than average-protein diets (protein 15%), despite similar weight reduction and no changes in fasting glucose at 24 months [41]. Other studies support the glucose-lowering effects of both diets. In the Prevención con Dieta Mediterránea (PREDIMED) study, a Mediterranean diet enriched with olive oil or nuts was associated with a 52% reduction in the incidence of diabetes over a 4-year follow-up period in participants without diabetes at baseline [10], and with lower need for glucose-lowering medications in those with diabetes [11]. A 6-month LC/high-protein diet produced a greater increase in glucose tolerance and insulin sensitivity compared with a standard diet in obese people with normal [25] and impaired [21] glucose tolerance, even despite similar weight loss. These studies are hardly comparable due to different patients’ characteristics, dietary prescriptions, study duration, and weight loss achieved. However, the improvement in fasting and postprandial hyperglycemia appears proportional to baseline plasma glucose levels, being negligible in patients with only minor glucose alterations, while the improvement in insulin sensitivity is consistent across studies and seems similar between the two diets, as also supported by our data.

Using an accurate C-peptide-based estimation of insulin secretion and clearance, we could characterize and compare for the first time the effects of each diet on insulin kinetics. Unexpectedly, given the lower peripheral insulin levels, we observed an enhancement of fasting and glucose-stimulated insulin secretion after the diet, whose extent was numerically greater for the LC diet than Med diet. The enhanced insulin secretion however did not result in higher plasma insulin levels as it was associated with a more than compensatory increase in insulin clearance at fasting and after the glucose load, which again tended to be greater with the LC diet. Endogenous insulin clearance mostly occurs in the liver, which accounts for the majority (up to 80%) of secreted insulin removal during its first-pass transit into the portal vein [42]. A reduced insulin clearance has been recently identified as a major pathogenetic mechanism of diabetes progression [43,44,45,46] and is typically associated with insulin-resistance [45,46,47], visceral obesity [47,48,49,50,51], and non-alcoholic fatty liver disease [46,47,51,52,53]. Therefore, the increase in insulin clearance observed in our study may be interpreted as a positive effect of the diet associated with the reduced body weight and whole-body/hepatic insulin resistance.

We hypothesized that an improvement in β-cell function would occur after the two diets. Indeed, dietary intake of monounsaturated fatty acids (MUFA) and n-3 polyunsaturated fatty acids (n-3 PUFA), which is especially high in the Mediterranean area, was associated with positive 7-year longitudinal changes in model-derived markers of β-cell function (i.e., β-cell glucose sensitivity and rate sensitivity) in a population-based study [54]. Furthermore, protein and amino acid consumption is known to enhance insulin secretion in both healthy [55,56,57,58] and diabetic individuals [59,60,61,62,63]. In line with this indirect evidence, previous randomized clinical trials in insulin-resistant subjects demonstrated an increase in surrogate markers of β-cell function after a Med diet [26] or LC diet [25]. Definite conclusions however could not be drawn from these studies, lacking an adequate characterization of β-cell function independent of insulin sensitivity and glucose levels. By using a mathematical model of β-cell function, here we demonstrated a significant and equal improvement in β-cell glucose sensitivity after the two dietary interventions. An increase in β-cell glucose sensitivity is graphically represented by a steeper dose-response function of the insulin secretion rates plotted against plasma glucose levels (Figure 2), meaning that the β-cell secretes more insulin secretion in response to the same glucose levels.

We acknowledge that this study has some limitations. We relied on self-reported dietary compliance, but we estimated that the adherence to the diet was adequate given that the actual weight loss was close to the predicted and that participants returned after 2 weeks for reinforcement of dietary prescriptions. While subjects on the LC/ high-protein diet lost more weight than those on the Med diet, we could not clearly identify group differences in glucometabolic parameters, possibly due to the small sample size. In fact, numerical differences emerged suggesting greater benefits of the LC diet on insulin secretion and clearance, which however did not reach statistical significance. Finally, our findings should not be generalized to subjects with overweight or low-grade obesity and in those with type 2 diabetes.

## 5. Conclusions

This study demonstrates that a LC/ high-protein diet is a successful short-term approach for weight loss in morbidly obese patients and a feasible alternative to the Med diet for its glucometabolic benefits, including rapid improvements in insulin resistance, insulin clearance and β-cell function. Further studies are needed to compare efficacy and safety of LC and Med diets in the long term.

## Figures and Tables

**Table 1:** Baseline clinical and metabolic characteristics of morbidly obese individuals randomized to a low-carbohydrate diet (LC) or Mediterranean (Med) diet.

	LC Diet (*n* = 17)	Med Diet (*n* = 15)	*p*
Age (years)	41.4 ± 10.5	46.9 ± 10.5	*ns*
Women (*n*; %)	12; 70.6	10; 66.7	*ns*
Systolic Blood Pressure (mmHg)	131 ± 9	143 ± 16	*0.07*
Diastolic Blood Pressure (mmHg)	79 ± 6	83 ± 7	*ns*
Body Mass Index (kg/m^2^)	48.9 ± 5.4	49.9 ± 8.8	*ns*
Body Weight (kg)	137.0 ± 19.3	136.0 ± 34.6	*ns*
Waist Circumference (cm)	131 ± 13	132 ± 15	*ns*
Waist-to-hip Ratio (ratio)	1.07 ± 0.14	1.07 ± 0.12	*ns*
Fat mass (%)	45.6 ± 3.2	44.1 ± 5.3	*ns*
BMR (calories/day)	2350 ± 490	2490 ± 655	*ns*
HbA1c (mmol/mol)	38 ± 5	38 ± 4	*ns*
Fasting glucose (mmol/L)	5.5 ± 0.5	5.7 ± 0.7	*ns*
Total cholesterol (mg/dL)	193 ± 41	178 ± 31	*ns*
HDL cholesterol (mg/dL)	51 ± 9	57 ± 15	*ns*
LDL cholesterol (mg/dL)	124 ± 33	93 ± 34	*0.08*
Triglycerides (mg/dL)	114 ± 31	125 ± 63	*ns*
Alanine Aminotransferase (U/L)	28 [16]	24 [31]	*ns*
Aspartate Aminotransferase (U/L)	19 [14]	19 [11]	*ns*
γ-Glutamyl Transferase (U/L)	22 [20]	22 [15]	*ns*
Creatinine (mg/dL)	0.76 ± 0.19	0.76 ± 0.20	*ns*
eGFR (ml min^−1^ 1.73 m^2^)	108 ± 21	102 ± 17	*ns*
Urea (mg/dL)	28.4 ± 5.9	30.7 ± 11.5	*ns*

Data are mean ± SD or median [interquartile range]. *p* values below 0.10 are shown.

**Table 2:** Changes in body weight, adiposity and basal metabolic rate (BMR) induced by a low-carbohydrate diet (LC) or Mediterranean (Med) diet in morbidly obese individuals.

	LC Diet (*n* = 17)		Med Diet (*n* = 15)		*p*		
	Day 0	Day 28	Day 0	Day 28	Diet	Time	Interaction
Body Mass Index (kg/m^2^)	48.9 ± 5.4	46.1 ± 5.1	49.9 ± 8.8	48.0 ± 8.4	*ns*	*<0.001*	*0.005*
Body Weight (kg)	137.0 ± 19.3	129.0 ± 18.0	136.0 ± 34.6	131.0 ± 32.4	*ns*	*<0.001*	*0.01*
Waist Circumference (cm)	131 ± 13	124 ± 11	132 ± 15	127 ± 14	*ns*	*<0.001*	*ns*
Waist-to-hip Ratio (ratio)	1.07 ± 0.14	0.92 ± 0.11	1.07 ± 0.12	0.93 ± 0.10	*ns*	*0.003*	*ns*
Fat mass (%)	45.6 ± 3.2	43.3 ± 2.9	44.1 ± 5.3	41.0 ± 5.7	*ns*	*<0.001*	*ns*
BMR (calories/day)	2350 ± 490	2169 ± 317	2490 ± 655	2234 ± 671	*ns*	*0.007*	*ns*

Data are mean ± SD. *p* values below 0.10 are shown.

**Table 3:** Changes in glucose and insulin metabolism induced by a low-carbohydrate diet (LC) or Mediterranean (Med) diet in morbidly obese individuals.

	LC Diet (*n* = 17)		Med Diet (*n* = 15)		*p*		
	Day 0	Day 28	Day 0	Day 28	Diet	Time	Interaction
**Glucose Control**							
Fasting Glucose (mmol/L)	5.5 ± 0.5	5.6 ± 0.5	5.7 ± 0.7	5.5 ± 0.7	*ns*	*ns*	*ns*
2-h Glucose (mmol/L)	7.3 ± 1.6	7.4 ± 1.8	7.0 ± 1.5	7.4 ± 2.1	*ns*	*ns*	*ns*
Mean Glucose (mmol/L)	7.3 ± 1.1	7.6 ± 1.2	7.3 ± 1.1	7.6 ± 1.7	*ns*	*ns*	*ns*
Fasting Insulin (pmol/L)	139 [105]	110 [75]	137 [99]	109 [67]	*ns*	*0.02*	*ns*
Mean Insulin (pmol/L)	473 [399]	411 [183]	447 [271]	508 [512]	*ns*	*ns*	*ns*
**Insulin Sensitivity**							
HOMA-IR (unit)	5.42 [5.22]	4.05 [2.94]	5.79 [5.54]	4.90 [4.64]	*ns*	*0.04*	*ns*
OGIS (ml min^−1^ m^−2^)	331 [90]	320 [97]	342 [93]	306 [141]	*ns*	*ns*	*ns*
**Beta-cell Function**							
Fasting ISR (pmol m^−2^ min^−1^)	119 [58]	135 [61]	123 [147]	130 [72]	*ns*	*0.0003*	*ns*
Total ISR (nmol/m^2^)	52 [25]	60 [23]	65 [48]	66 [33]	*ns*	*0.05*	*ns*
ISR@5 (pmol min^−1^ m^−2^)	105 [59]	106 [48]	106 [90]	119 [97]	*ns*	*ns*	*ns*
β-GS (pmol min^−1^ m^−2^ mM^−1^)	57 [80]	82 [38]	64 [53]	86 [65]	*ns*	*0.03*	*ns*
β-RS (nmol m^−2^ mM^−1^)	1000 [898]	898 [1067]	1187 [1216]	1019 [1055]	*ns*	*ns*	*ns*
Potentiation Factor (ratio)	1.15 [0.24]	1.15 [0.48]	1.42 [0.71]	1.41 [0.76]	*ns*	*ns*	*ns*
**Incretin Hormones**							
GLP-1 AUC (nmol/L × min)	9.5 [4.8]	7.3 [5.2]	7.6 [4.6]	6.3 [3.5]	*ns*	*0.002*	*ns*
GIP AUC (nmol/L × min)	36.6 [28.9]	35.8 [25.8]	43.2 [31.6]	38.7 [42.2]	*ns*	*ns*	*ns*
**Insulin Clearance**							
Fasting Clearance (l min^−1^ m^−2^)	0.92 [0.50]	1.26 [0.56]	1.02 [0.66]	1.15 [0.46]	*ns*	*0.005*	*0.06*
OGTT Clearance (l min^−1^ m^−2^)	0.62 [0.29]	0.81 [0.30]	0.74 [0.33]	0.71 [0.29]	*ns*	*0.05*	*0.06*

Data are mean ± SD or median [interquartile range]. *p* values below 0.10 are shown.

## Data Availability

The data presented in this study are available on request from the corresponding author.
